# Effect of Vitamin C on the Antioxidant and Immune Response of Male White Shrimp (*Penaeus vannamei*) Broodstock

**DOI:** 10.3390/antiox14080988

**Published:** 2025-08-12

**Authors:** Grecia Montalvo, Sarabí Caballeros, Karla Escalante, Alvaro Barreto, Martín Arenas, Gabriela Gaxiola

**Affiliations:** 1 Unidad Multidisciplinaria de Docencia e Investigación (UMDI) Sisal, Facultad de Ciencias, Universidad Nacional Autónoma de México (UNAM), Yucatán, Hunucmá 97351, Mexico; greciamontalvofernandez@gmail.com (G.M.); karla.escalante@ciencias.unam.mx (K.E.); alvarobarreto09@gmail.com (A.B.); 2 Instituto Tecnológico de Conkal, Yucatán, Mérida 97345, Mexico; sary-26@outlook.com; 3 Departamento de Recursos del Mar, Centro de Investigación y de Estudios Avanzados del Instituto Politécnico Nacional, Unidad Mérida, Km 6 Carretera Antigua a Progreso, Cordemex, Yucatán, Mérida 97319, Mexico; martin.arenas@cinvestav.mx

**Keywords:** oxidative stress, superoxide dismutase, catalase, sperm quantity and quality, shrimp, vitamin C

## Abstract

This study evaluated the effect of vitamin C (L-ascorbyl-2-polyphosphate) on the physiological condition, biochemical antioxidant activity, immune responses, and gene expression in the reproductive tract, as well as on sperm quantity and quality in male white shrimp *Penaeus vannamei* broodstock. Four diets containing 42.5% protein, 11.5% lipids, and 23.5% carbohydrates were formulated with L-ascorbyl-2-polyphosphate as a source of vitamin C at the following concentrations: 0.016 g/kg (Basal), 0.322 g/kg (A), 0.628 g/kg (B), and 0.934 g/kg (**C**). Shrimp fed diet **C** exhibited the highest SOD and CAT activity and serum cholesterol levels, but the lowest expression of hemocyanin (Hemo) mRNA transcripts (*p* ˂ 0.05). Shrimp fed diet A showed the highest Hemo mRNA expression and phenoloxidase (PO) activity, while those fed diet B had the highest serum triglyceride levels (*p* ˂ 0.05). In contrast, shrimp fed diets A and B exhibited the lowest serum cholesterol levels (*p* ˂ 0.05). There were no differences in sperm quality between the diets. In relation to sperm quantity, the shrimp fed diet B had the highest sperm cell count (2,750,000 cel/mL), and those fed diet A had the lowest (585,000 cel/mL) (*p* ˂ 0.05). These results indicate that vitamin C influences the reproductive aspects of male *P. vannamei* broodstock. A dietary inclusion level of 0.628 g/kg promotes optimal physiological, oxidative stress, and immunological conditions for increased sperm cell production, whereas an excessive level may promote oxidative stress.

## 1. Introduction

Vitamins play an important role in promoting growth and maintaining the overall health of shrimp; however, their specific dietary requirements remain poorly defined [1]. Among these, vitamin C (ascorbic acid) is particularly important, as shrimp are incapable of synthesizing it de novo [2]. As a potent antioxidant, vitamin C works synergistically with vitamin E to inhibit lipid peroxidation [3]. However, when present in excess, vitamin C may exhibit pro-oxidative activity by reducing free metal ions, which can catalyze the formation of reactive oxygen species [4].

Adequate supplementation of dietary vitamin C improves survival, antioxidant capacity, and non-specific immune response in postlarvae and juvenile shrimp [5,6,7,8,9,10]. With respect to broodstock shrimp, research on vitamin C requirements remains limited, despite the global importance of shrimp aquaculture [1]. In wild broodstock of *Penaeus vannamei*, the gonads have been identified as the primary storage site for vitamin C [11]. In contrast, a lack of dietary vitamin C supplementation in domestic-broodstock *Penaeus japonicus* has been shown to negatively impact gonadal development [12]. These findings suggest the potential link between vitamin C and reproductive performance.

Achieving reproductive success in shrimp in captivity requires adequate nutrition for both female and male broodstock; however, males have received little attention in comparison to females [13]. To date, the shrimp broodstock feeding protocols rely on fresh frozen food supplemented with artificial diets to ensure an adequate supply of essential nutrients such as vitamins, typically provided through a vitamin premix [14,15]. However, it remains unknown whether these vitamin premixes provide sufficient dietary supplementation of specific vitamins, such as vitamin C. Recently, in male *Penaeus brasiliensis* broodstock, the inclusion of dietary L-ascorbyl-2-phosphate as a source of vitamin C at 0.934 g/kg, compared to 0.016 g/kg in the control diet (vitamin premix), improved its physiological status [16].

*Penaeus vannamei*, commonly known as Pacific white shrimp, is the most important species in aquaculture shrimp, accounting for over 50% of worldwide production [17]. However, one of the major challenges in *P. vannamei* aquaculture is the melanization of the male broodstock reproductive tract, which results in reduced sperm quality and consequently affects fertilization rates and nauplii output [18]. This physiological disorder has frequently been associated with oxidative stress related to nutritional factors [19,20]. The aim of this study is to evaluate the effect of L-ascorbyl-2-phosphate, included in feed, as source of vitamin C on the physiological condition, immune and antioxidant responses in the reproductive tract, and sperm quality of *P. vannamei* male broodstock under culture conditions.

## 2. Materials and Methods

### 2.1. Experimental Diets

Four diets containing 42.5% protein, 11.5% lipids, and 23.5% carbohydrates were formulated with vitamin C (L-ascorbyl-2-polyphosphate, Stay-C^®^ 35 ROVIMIX^®^, DSM de Mexico SA de CV, El Salto, Mexico) at the following concentrations: 0.016 g/kg (Basal diet), 0.322 g/kg (A), 0.628 g/kg (B), and 0.934 g/kg (C). The diets were manufactured according to Montalvo et al. [16].

### 2.2. Dietary Proximal Analysis

Protein content was determined using an elemental analyzer (ECS-4010, Costech, Raleigh, NC, USA). Lipid content was determined by the Golden System method with hexane [21]. Ash content was determined by incineration for six hours at 550 °C in a muffle furnace. Moisture content was assessed by drying samples for 120 h at 60 °C in an oven [22]. To determine vitamin C content, the samples were dissolved in HPLC-grade methanol in a 50 mL calibrated brown flask, sonicated for 40 min at 24 °C, and filtered through a nylon membrane with a 0.45 µm pore size (Z290815, Sigma-Aldrich, St. Louis, MO, USA); the resulting solution was read at 245 nm. Sodium L-ascorbyl-2-phosphate (49752, Sigma-Aldrich) was used as the standard [23].

### 2.3. Experimental Procedures

Broodstock males of *P. vannamei* (mean ± standard deviation = 20 ± 3.6 g initial wet weight) were obtained from UMDI Sisal, Facultad de Ciencias, UNAM, Yucatán, México [18].

The trial was conducted in a recirculation water system equipped with twelve circulate 500 L fiberglass tanks and a 1200 L reservoir. Seven male shrimp were randomly assigned to each tank, with three replicate tanks per dietary treatment. The system operated with a 1 HP pump (Pentair SuperFlo^®^, Minneapolis, MN, USA), and the water was treated using an ultraviolet sterilizer (BAP4011, Novem, Luxembourg), a homemade skimmer, and a sand and biological filter (EF-1200U, Resun, Shenzhen, China). Each tank was maintained at a controlled temperature using titanium aquarium heaters (ViaAqua Heaters Titanium, Nantes, France) and supplied with continuous aeration (CL K04-MS 1 HP, FPZ, Saukville, WI, USA). The water quality parameters during the trial were as follows: dissolved oxygen (6.1 ± 1.0 mg/L), temperature (28.1 ± 0.4 °C), pH (7.79 ± 0.05), salinity (35 ppm), and total ammonia nitrogen (0.11 mg/L). The broodstock males were fed ad libitum at 08:00, 14:00, and 20:00 h. After each feeding, the tank bottoms were siphoned to remove any uneaten feed.

### 2.4. Sample Collection

The experimental assay lasted 30 days. Four unfed (starved) shrimp were randomly selected from each tank; there were three tanks for each diet, so a total of 12 samples were taken for each diet. Hemolymph was collected from the ventral sinus using a 3 mL BD Plastipak™ syringe pre-rinsed with SIC-EDTA (450 mmol NaCl, 10 mmol KCl, 10 mmol Hepes, 10 mmol EDTA Na_2_), pH 7.3. The hemolymph was diluted with SIC-EDTA at a 1:2 ratio (200 µL of hemolymph to 400 µL of SIC-EDTA) and immediately stored at 4 °C for 1 to 3 days until processing.

The reproductive system was dissected and divided into two halves (including testis, spermatic conduct, and terminal ampule) for biochemical and genetic analysis, respectively. The half designated for biochemical analysis was frozen in liquid nitrogen and stored at −80 °C. The other half, intended for genetic analysis, was preserved in RNAlater^®^ (R0901, Sigma-Aldrich) and stored at −80 °C. Prior to freezing the sample for biochemical analysis, the spermatophore was extruded from the terminal ampoule for sperm quality evaluation. The study was conducted according to the guidelines of the bioethics subcommittee of the Ethics and Scientific Responsibility Commission of the Facultad de Ciencias, UNAM (Folio: PI_23_04_2025_Gaxiola).

### 2.5. Hemolymph Biochemical Analysis

Hemolymph samples preserved in SIC-EDTA were centrifuged for 3 min at 6 000 rpm at 4 °C (5420R—Eppendorf, Hamburg, Germany). The resulting supernatant was used to measure triglycerides, cholesterol, and glucose levels using commercial assay kits, following the manufacture’s instruction (TGML-0427, CHSL-4150, GPSL-0507—ELITechGroup, Puteaux, France). Total soluble protein was evaluated with Bio-Rad Protein Assay (5000006, BIORAD, Hercules, CA, USA); a curve of bovine serum albumin (B2064, Sigma-Aldrich) used as the standard [24].

For phenoloxidase (PO) activity, the hemocyte cell pellets obtained from centrifugation were lysed using 100 mmol cacodylate buffer, pH 7 (C0125, Sigma-Aldrich). To measure PO activity, 10 µL of samples were combined with 250 µL of 15 mmol L-DOPA (D9628, Sigama-Aldrich) and incubated for 20 min at 25 °C. Enzymatic activity was reported as absorbance per minute per mg of soluble protein [25].

### 2.6. Reproductive System Biochemical Analysis

Reproductive system tissue was homogenized using an IKA T18 ULTRA-TURRAX^®^ homogenizer in 50 mM tris buffer (252859, Sigma-Aldrich), pH 7.4. The homogenate was divided into two portions for oxidative stress and antioxidant enzyme analysis, respectively. The portions designed for antioxidant enzyme analysis were centrifuged for 25 min at 10,000 rpm at 4 °C. The resulting supernatant was used to measure the activities of antioxidant enzymes: superoxide dismutase (SOD), catalase (CAT), and glutathione peroxidase (GPx).

The SOD assay is based on the rate of reduction of cytochrome c by the superoxide anion, which is measured at 550 nm. The assay was performed by incubating 10 µL of sample in 180 µL of 50 mmol phosphate buffer (pH 7.8) containing 5 µmol EDTA (E9885, Sigma-Aldrich), 50 µmol hypoxanthine (H9377, Sigma-Aldrich), and 20 µmol cytochrome c (C2506, Sigma-Aldrich). The reaction was started by adding 10 µL of xanthine oxidase (0.2 U/mL) (X4875, Sigma-Aldrich), followed by 5 min incubation. One unit of SOD activity was defined as the amount of enzyme that inhibits the rate of reduction of cytochrome c by 50% at a 20 µmol concentration [26,27].

The CAT assay is based on the formation of a yellow complex between ammonium molybdate (277908, Sigma-Aldrich) and hydrogen peroxide (H1009, Sigma-Aldrich), which is measured at 405 nm. The assay was performed by incubating 10 µL of sample in 100 µL of 50 mmol phosphate buffer (pH 7.4) containing 8.8 mol hydrogen peroxide for 3 min at 25 °C. The reaction was stopped by adding 100 µL of 200 mmol of ammonium molybdate. A control assay was carried out by incubating 10 µL of sample in 100 µL of 50 mmol phosphate buffer (pH 7.4) for 3 min at 25 °C, after which the reaction was stopped by adding 100 µL of 200 mmol of ammonium molybdate. One unit of CAT activity was defined as the number of micromoles of hydrogen peroxide discomposed per minute [28].

The GPx assay is based on the oxidation rate of NADPH measured at 340 nm. The assay was performed by incubating 10 µL of sample in 120 µL of 50 mmol tris buffer (pH 7.6) containing 0.14 mmol NADPH (10107824001, Sigma-Aldrich), 0.1 mM EDTA, 0.1 mmol of glutathione (G4251, Sigma-Aldrich), and 1 U glutathione reductase (G3664, Sigma-Aldrich). The activity was initiated by adding 15 µL of 0.2 mmol t-butyl hydroperoxide (458139, Sigma-Aldrich), followed by 5 min incubation. One unit of GPx activity was defined as the number of micromoles of NADPH oxidized per minute [26]. Enzymatic activities were reported as mg of soluble protein (specific activity).

For oxidative stress, lipid peroxidation (LPO) was evaluated using the FOX method. The assay is based on the oxidation of Fe^2+^ to Fe^3+^, which then binds with xylenol orange, with the resulting complex measured at 560 nm. The uncentrifuged homogenate was mixed with methanol in a 1:1 ratio and centrifugated at 10 000 for 5 min. LPO was determined following the methodology described by Banerjee et al. [29], with tert-butyl hydroperoxide (458139, Sigma-Aldrich) used as a standard.

### 2.7. Real-Time Quantitative Polymerase Chain Reaction (RT-qPCR)

Total RNA was extracted from 0.1 g of sample using the TRIzol protocol (Trizol^®^, Sigma-Aldrich^®^, Toluca, Mexico). RNA integrity was determined by 1% agarose gel electrophoresis, and its purity was quantified at 260/280 nm with a Nanodrop spectrophotometer (ND-2000, Thermo Scientific, Wilmington, DE, USA). For cDNA synthesis, the samples (10 µL of RNA 300 ng/µL) were treated with DNAse I to remove any DNA contamination in a 10 µL reaction volume. The reaction was conducted at 37 °C for 30 min; then, 1 μL EDTA was added to the samples and incubated at 65 °C for 10 min.

The cDNA synthesis was performed in 20 µL: 10 µL total RNA 300 ng/μL, 2 μL 10 × reverse transcriptase (RT) buffer + MgCl_2_, 0.8 μL 10 mM dNTP, 2 μL 10 × RT random primers, 1 μL U-reverse transcriptase (Sigma-Aldrich, St. Louis, MO, USA), 1 μL RNAsa inhibitor, and 3.2 µL nuclease-free water. The PCR program consisted of the following steps: 25 °C for 10 min, 37 °C for 120 min, 85 °C for 5 min, and finally at 4 °C.

The BIO-RAD^®^ CFX96 thermocycler was used to carry out the RT-qPCR analysis using Universal SYBR^®^ Green Supermix (172–5121, Bio-Rad^®^, Philadelphia, PA, USA). The reaction was carried out in a 15 µL reaction volume: 7.5 μL SYBR green super mix, 0.5 μL of each forward and reverse primer, 4.5 μL pyrogen-free water, and 2 μL cDNA. The RT-qPCR program consisted of the following steps: 95 °C for 3 min with 35 cycles, 95 °C for 30 s, and 60 °C for 30 s.

For each primer pair (Table 1), the RT-qPCR efficiency was calculated using a standard curve produced by the serial dilution of the cDNA pool. The 2^−ΔΔCt^ method was employed to calculate the relative expression of the genes [30]. The β-actin gene was used as an internal control. The *α2 macroglobulin* (A2M), *hemocyanin* (Hemo), *penaeidin 3* (*penaeidin*), ProPO, SOD, CAT, and GPx primers were obtained from Aguilera-Rivera et al. [31] and Montalvo et al. [32] (Table 2).

### 2.8. Spermatic Quantity and Quality

The extruded spermatophores were homogenized in calcium-free saline solution using a pellet pestle in a 5 mL Eppendorf tube. Then, 900 µL of the resulting homogenate was filtered and placed in a 1.5 mL Eppendorf tube with 0.1 mL of trypan blue (1% *w*/*v* in calcium-free solution). After 10 min incubation, the samples were read in a BLAUBRAND^®^ Neubauer chamber (Wertheim, Germany). Sperm cells were classified as normal, abnormal, or dead [33].

### 2.9. Statistical Analyses

Sperm count was analyzed using a negative-binomial mixed model [34], with parameter estimation performed through maximum likelihood estimation. The experimental unit was the tank, which was considered a random effect. Enzymatic activity, relative gene expression, and percentage data were modeled using generalized least squares, incorporating a variance structure per treatment. Percentage data and relative gene expression were transformed using arcsine square root and the base-ten logarithm, respectively.

For model fitting, the observed data were compared with fitted values. For model validation, Pearson’s residual plots were compared against the fitted values and each treatment. Significance was determined at *p* ˂ 0.05. When significant differences were found, post hoc tests were conducted with a Tukey’s test adjustment [35]. The data were statistically analyzed using R software 4.02 [36].

## 3. Results

### 3.1. Hemolymph Biochemical Analysis

**Figure 1:**
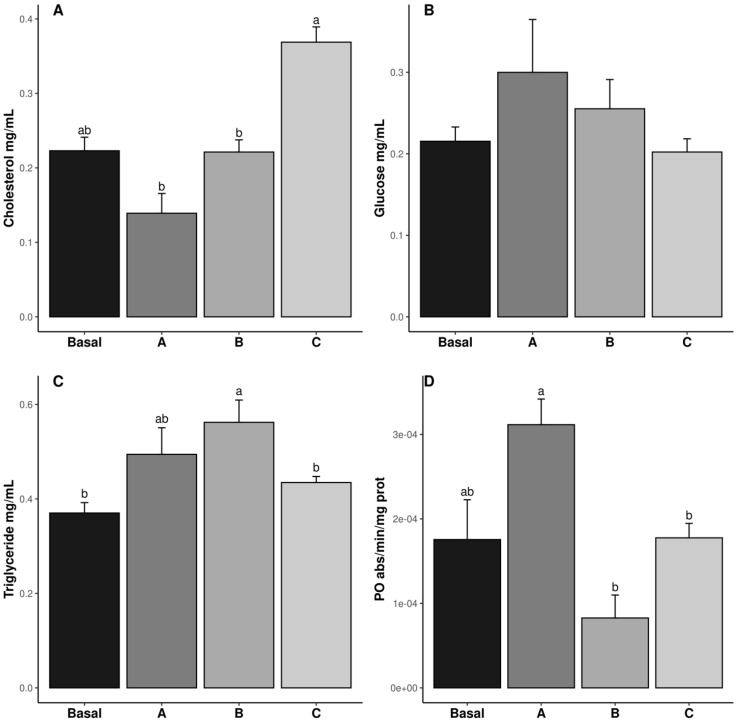
Hemolymph metabolite concentrations (cholesterol, (**A**); glucose, (**B**); Triglycerides, (**C**); PO enzymatic activity, (**D**)); in male *Penaeus vannamei* broodstock fed with different inclusion levels of vitamin C in the diet: Basal (0.016 g/kg), A (0.322 g/kg), B (0.628 g/kg), and C (0.934 g/kg). The results are expressed as mean ± SE (*n* = 3). Different letters in the superscripts indicate significant differences between treatments (*p* < 0.05).

The cholesterol, triglyceride (TG), glucose, and PO results are shown in Figure 1. The cholesterol levels were the highest in the shrimp fed diet C, whereas those fed diets A and B exhibited the lowest values (*p* ˂ 0.05). The cholesterol value in the Basal diet was similar to all treatments (*p* ˃ 0.05) (Figure 1A). The TG levels were highest in the shrimp fed diet B, while those fed the Basal and C diets exhibited the lowest values (*p* ˂ 0.05). The TG content in the shrimp fed diet A was similar to the other treatments (*p* ˃ 0.05) (Figure 1C). The PO values in the shrimp fed diets B and C were lower than in those fed diet A (*p* ˂ 0.05), whereas those fed the Basal diet were similar to all treatments (*p* ˃ 0.05) (Figure 1D). Glucose levels were not affected by vitamin C inclusion in the diet (*p* ˃ 0.05) (Figure 1B).

### 3.2. Biochemical Analysis and Relative Expression of Genes of Antioxidant Enzyme System in the Reproductive System

**Figure 2:**
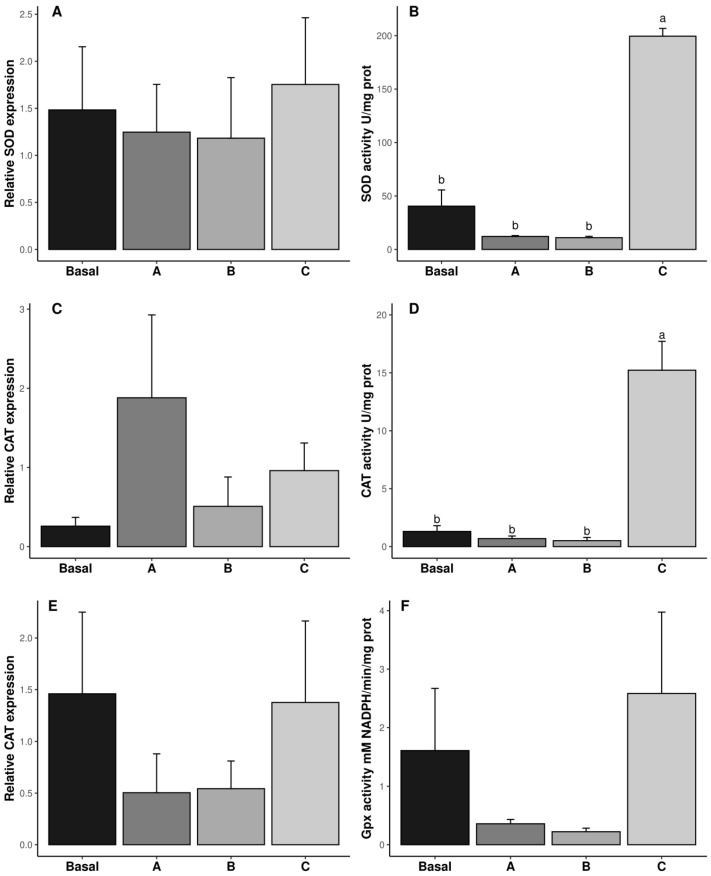
Relative gene expression (**A**,**C**,**E**) and biochemical activity (**B**,**D**,**F**) of antioxidant responses in reproductive tract of male *Penaeus vannamei* broodstock fed with different inclusion levels of vitamin C in the diet: Basal (0.016 g/kg), A (0.322 g/kg), B (0.628 g/kg), and C (0.934 g/kg). The results are expressed as mean ± SE (*n* = 3 pooled samples). Different letters in each graphic indicate significant differences between treatments.

The specific enzymatic activity and gene expression of SOD, CAT, and GPx are shown in Figure 2. Dietary vitamin C concentrations did not affect the transcription of SOD, CAT, and GPx (*p* ˃ 0.05, Figure 2A,C,E). However, regarding SOD and CAT, specific enzyme activities were significantly affected at the translation level, being highest in diet C (*p* ˂ 0.05, Figure 2B,D), whereas GPx specific activity was not significantly affected by dietary vitamin C. In the same way, LPO was not affected by vitamin C inclusion in the diet (*p* ˃ 0.05, Figure 2E and Table 2).

**Figure 3:**
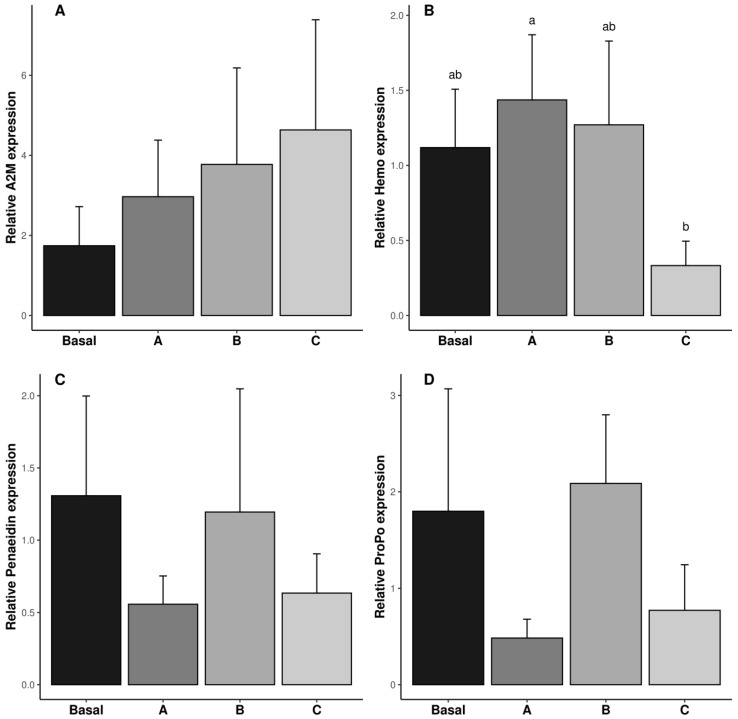
Relative gene expression of immune responses (A2M: (**A**)); Penaeidin (**B**); Hemo (**C**) and poPO (**D**) in reproductive tract of male *Penaeus vannamei* broodstock fed with different inclusion levels of vitamin C in the diet: Basal (0.016 g/kg), A (0.322 g/kg), B (0.628 g/kg), and C (0.934 g/kg). The results are expressed as mean ± SE (*n* = 3 pooled samples). Different letters in each graphic indicate significant differences between treatments.

The relative expressions of *A2M*, *Hemo*, *Penaeidin*, and ProPO genes in the reproductive tract are shown in Figure 3. The relative expression of the *Hemo* gene was the lowest in the shrimp fed diet C (*p* ˂ 0.05). The relative expression of the *A2M*, *Penaeidin*, and *ProPO* genes was not affected by vitamin C inclusion in the diet (*p* ˃ 0.05, Figure 3A,C,D).

### 3.3. Spermatic Quantity and Quality

**Figure 4:**
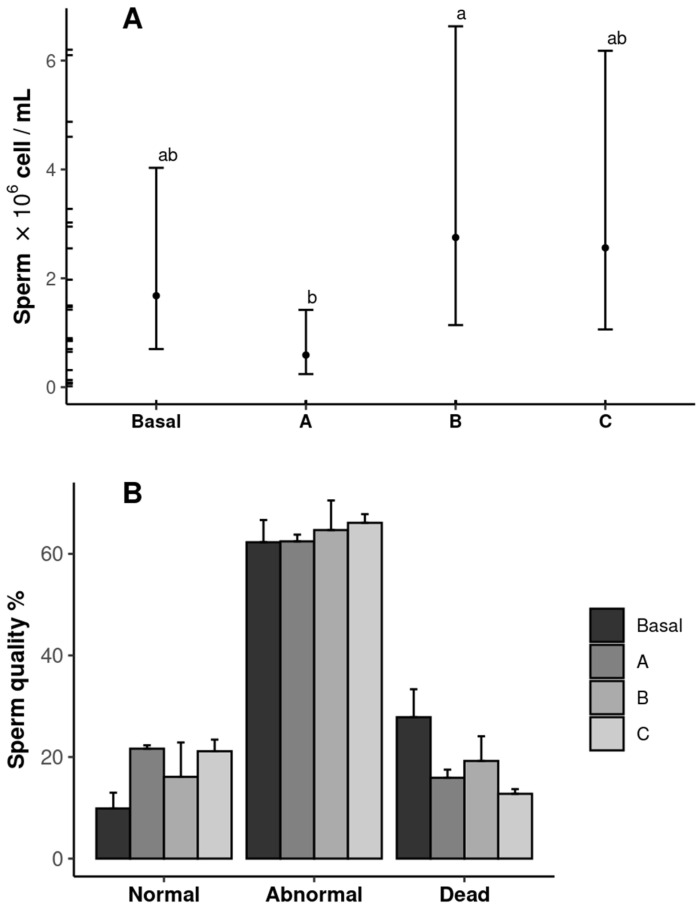
Sperm quantity (**A**) and quality (**B**) of male *Penaeus vannamei* broodstock fed with different inclusion levels of vitamin C in the diet: Basal (0.016 g/kg), A (0.322 g/kg), B (0.628 g/kg), and C (0.934 g/kg). The results are expressed as mean ± SE (*n* = 3). Significant letters indicate significant differences between treatments.

The sperm quantity and quality results are shown in Figure 4. The shrimp fed diet B (2.75 × 10^6^ cells mL^−1^) exhibited the highest sperm count, whereas those fed diet A presented the lowest value (*p* ˂ 0.05). The sperm count in the shrimp fed the Basal and C diets was similar in all treatments (*p* ˃ 0.05) (Figure 4A). No differences were observed in the sperm quality among the treatments (*p* ˃ 0.05) (Figure 4B).

## 4. Discussion

The optimal vitamin C requirement, specially the male broodstock, has not been clearly established for most farmed species of penaeid shrimp, which may vary since different criteria have been used. In other words, it depends on the response variables [37]. Many authors have studied vitamin C requirements based on weight gain and survival. This study used variables related to the physiological conditions, immune and antioxidant responses in the reproductive tract, and sperm quality of male *P. vannamei* broodstock.

The results indicate that adding vitamin C to the diet of *P. vannamei* broodstock males has a positive effect, but up to a certain limit. Diet (B), supplemented with 0.628 g/kg, seemed to generate a good physiological state as it increased cholesterol metabolism and the relative expression of hemocyanin transcripts, as well as reducing the activity of the endogenous antioxidant system (SOD, CAT, and GPx). Additionally, this diet resulted in an increase in the amount of sperm with respect to other treatments (*p* < 0.05).

Comparing these results with those obtained when we added the same amount of vitamin C to the diet of breeding males of *P. brasiliensis* shows that a higher amount of vitamin C is required to achieve a good physiological state of individuals; at 0. 934 g/kg, SOD activity is reduced [16]. However, this amount may be excessive for *P. vannamei* because it increases endogenous antioxidant system activity (SOD and CAT), which participates in defense mechanisms and may indicate some physiological stress.

### 4.1. Hemolymph Biochemical Analysis

Vitamin C (ascorbic acid) is an essential nutrient for shrimp as, unlike humans, they cannot synthesize it and it must be supplied through their diet. However, an excess of this essential nutrient could induce a pro-oxidant effect [38]. Cholesterol is a vital structural component of cell membranes and a precursor of steroid hormones. However, it also contributes to the formation of oxysterols, oxidative derivates of cholesterol that act as pro-oxidants. In mammals, the protective role of vitamin C against cholesterol oxidation—which leads to the formation of oxysterols—has been well documented, as it facilitates the enhanced metabolic clearance of cholesterol [39,40]. In the present study, the lowest plasma cholesterol level was observed in shrimp fed diets A (0.322 g/kg) and B (0.628 g/kg), suggesting an increase in cholesterol metabolism compared to those fed the Basal and C diets. The elevated plasma cholesterol concentration in shrimp fed the Basal and C diets may be associated with low recognition by cholesterol receptors, potentially due to peroxidation modification resulting from either an excess or deficiency of vitamin C.

In the catabolism of triglycerides, vitamin C participates in the biosynthesis of carnitine, which facilitates the transport of fatty acids across mitochondrial membranes for their beta oxidation [41,42]. In mammals, vitamin C deficiency is associated with hypertriglyceridemia [43]; however, *P. vannamei* fed diet B exhibited the highest plasma triglyceride levels, an opposite effect to that observed for cholesterol. The shrimp fed the diet C exhibited the lowest serum triglyceride levels, indicating an increase in the metabolism of this metabolite.

In invertebrates, PO is considered an indicator of health status [44]. The primary role of this enzyme is to catalyze the melanization process by oxidizing phenols to produce quinones, which are responsible for melanin formation [45]. Melanization serves as a major defense mechanism and plays a role in wound healing [46]. In the present study, PO activity decreased in shrimp fed diets with higher vitamin C inclusion levels (B and C). These findings suggest that the antioxidant properties of vitamin C may have suppressed PO activity by mitigating oxidative stress, thereby reducing the stimuli necessary to trigger the activation of the ProPO system. In contrast, the exposure of isolated hemocytes from *Portunus trituberculatusto* parasites resulted in increased PO activity in response to higher levels of vitamin C [47]. This indicates that the effect of vitamin C on PO activation mechanisms depends on the physiological status of the organism.

### 4.2. Biochemical Analysis and Relative Expression of Genes in the Reproductive System

SOD, CAT, and GPx enzymes are considered the first line of antioxidant defense. In the present study, the expression of mRNA transcripts of these enzymes did not show significant differences among treatments; however, differences were observed in their activity. The activity of SOD and CAT was upregulated in shrimp fed the diet **C**, whereas no significant differences were observed in GPx activity. The SOD enzyme catalyzes the dismutation of superoxide anion to hydrogen peroxide and oxygen; then, the hydrogen peroxide is catalyzed to water and oxygen by CAT, and water by GPx, respectively (Hong and Park, 2021) [48]. The difference between CAT and GPx is their affinity by substrate (hydrogen peroxide): the first enzyme has a higher Km (Michael constant) (1.1 mol) than the second (1 µmol) [48,49]. The higher activity of SOD and CAT enzymes in treatment C indicates acute oxidative stress; this could be associated with a pro-oxidant effect from an excess of vitamin C. It is also important to point out that vitamin C induced effects at biochemical level, differing from *Penaeus brasiliensis* [16].

### 4.3. Relative Gene Expression of Immune-Related System

Increasing the amount of vitamin C from the Basal diet to diet B enhanced the differential expression of mRNA transcripts of hemocyanin (*Hemo*), an oxygen-carrying protein found in the hemolymph. Oxygen, as an oxidizing molecule, is involved in the production of energy; however, it also generates reactive intermediate products or free radicals that, depending on their oxidizing degree, can damage biological molecules, such as proteins, lipids, and nucleic acids [50]. Hemocyanin helps prevent the formation of reactive oxygen species by transporting it in its active center coupled to two copper molecules. Hemocyanin, in its oxidized form or in the presence of certain stimuli (such as infections or environmental stress), can bind or sequester free radicals directly, acting as a scavenger molecule. In contrast, shrimp fed diet C exhibited decrease levels of Hemo mRNA transcripts compared to those fed diet A, suggesting a reduced capacity for oxygen transport. This reduction may be associated with the elevated activity of SOD and CAT enzymes observed in the same treatment. The results in this study indicate that vitamin C functions as an immune system enhancer up to a certain limit, from which a pro-oxidant effect is presented. This activity was previously proposed by Lee and Shiauf [51] for *P. monodon*, although it should be noted that vitamin requirements are species-specific, so *P. monodon* and *P. vannamei* may have different requirements. Another factor that may influence the comparison of their results is that the study with *P. monodon* involved juvenile individuals and in our study, we worked with breeding males.

### 4.4. Spermatic Quantity and Quality

The observed differences in sperm cell quantity among the treatments indicate that vitamin C plays a significant role in the reproductive aspects of male *P. vannamei* broodstock. On the other hand, it is noteworthy that no significant differences were observed in sperm cell quality, despite the differences in quantity, as previously reported in *P. brasiliensis* [16]. These findings suggest that the quality of sperm cells may be influenced by other dietary nutrients. In terms of sperm cell production, shrimp fed diet B exhibited the highest sperm cell count, whereas those fed diet A showed the lowest. However, both groups displayed similar physiological, oxidative stress, and immunological responses, indicating that 0.322 g/kg (diet A) is insufficient to effectively stimulate sperm cell production. In contrast, the decrease in sperm cell production in the shrimp fed diet C could be associated with oxidative stress induced by a pro-oxidant effect of 0.934 g/kg vitamin C [52,53].

## 5. Conclusions

Vitamin C influences the reproductive aspects of male *P. vannamei* broodstock. In this study, a dietary vitamin C inclusion level of 0.628 g/kg (diet B) reduced oxidative stress and improved physiological and immunological conditions, leading an increase in sperm cell production. Additionally, the results of this study support the proposition that vitamin requirements are species-specific. While an inclusion level of 0.934 g/kg (diet **C**) of vitamin C is adequate to maintain a healthy physiological state in male *P. brasiliensis* broodstock [14], this amount appears excessive for *P. vannamei*, leading to a pro-oxidant effect. The absence of significant differences observed in the transcripts of antioxidant system genes in the reproductive tract of male *P. vannamei* allow us to conclude that the effect of dietary vitamin C occurs as biochemical adaptation expressed through antioxidant system enzyme activities. The results of this study are highly relevant for shrimp farming, as they demonstrate that maintaining broodstock with good sperm quality can improve fertilization rates and potentially increase nauplii production. Further studies are needed to confirm the role of vitamin C in controlling oxidative stress in female shrimp to optimize reproductive outcomes.

## Figures and Tables

**Table 1:** Oligonucleotides used for amplification of antioxidant and immune-related genes *Penaeus vannamei*, including *beta actin* (*β-actin*), *superoxide dismutase* (*SOD*), *catalase* (*CAT*), *glutathione peroxidase* (*Gxp*), *alpha 2 macroglobulin* (*a2M*), *prophelonoxidase* (*ProFo*), *hemocyanin* (Hemo), and *penaeidin 3* (*Penaeidin*).

Gene	Sequence		Size pb	GenBank Accession No.
	Forward	Reverse		
*β-actin*	5′-TGTGTGACGACGAAGTAGCC-3′	5′-TGGTCGTGAAGGTGTAACCA-3′	142	AF300705
*SOD*	5′-AGCTTACATCTCCATCCTGG-3′	5′-ATCTGAGGACTGACTGTGC-3′	189	DQ298207
*CAT*	5′-ACTCCCATTGCTGTTCGT-3′	5′-ATCCCAATTTCCTTCTTCTG-3′	195	JX162772
*GPx*	5′-AGTCGATGTCAACGGGTCAAC-3′	5′-GCTGAACCTCTTAAACGGCTG-3′	180	AY973252
*a2M*	5′-GTTTCCATCACCGCCTCA-3′	5′-ACCTTATCCTGCGGTGCCA-3′	227	EF182745
*ProFo*	5′-ACCGTACAAGGAAGAGGAAC-3′	5′-TCTCGCAGGTCGTTGTTGAT-3′	222	AY723296
*Hemo*	5′-GTCTTAGTGGTTCTTGGGCTTGTC-3′	5′-GGTCTCCGTCCTGAATGTCTCC-3′	124	KJ151291
*Penaeidin*	5′-CTGGTCTTCTTGCCTCCTT-3′	5′-ATATCCCTTTCCCACGTGAC-3′	121	AF390139

**Table 2:** Lipid peroxidation (LPO, (nmol/mg tissue) in the reproductive tract of male *Penaeus vannamei* broodstock fed with different inclusion levels of vitamin C in the diet: Basal (0.016 g/kg), A (0.322 g/kg), B (0.628 g/kg), and C (0.934 g/kg). The results are expressed as mean ± SE (*n* = 3 pooled samples).

Treatment	Mean	SE
Basal	0.058	0.00585684
A	0.04	0.01921331
B	0.03	0.01964186
C	0.047	0.03985511

## Data Availability

The data used to support the findings of this study are available from the corresponding author upon reasonable request.
